# Prevalence and potential risk factors of self-reported diabetes among elderly people in China: A national cross-sectional study of 224,142 adults

**DOI:** 10.3389/fpubh.2022.1051445

**Published:** 2022-12-16

**Authors:** Xing Hu, Lingbing Meng, Zhimin Wei, Hongxuan Xu, Jianyi Li, Yingying Li, Na Jia, Hui Li, Xin Qi, Xuezhai Zeng, Qiuxia Zhang, Juan Li, Deping Liu

**Affiliations:** ^1^Health Service Department of the Guard Bureau of the Joint Staff Department, Beijing, China; ^2^Graduate School of Peking University Health Science Center, Beijing, China; ^3^Department of Cardiology, Beijing Hospital, National Center of Gerontology, Institute of Geriatric Medicine, Chinese Academy of Medical Sciences, Beijing, China; ^4^China Research Center on Aging, Beijing, China; ^5^Institute of Psychology, Chinese Academy of Sciences, Beijing, China

**Keywords:** prevalence, self-reported, diabetes, elderly, national cross-sectional study

## Abstract

**Aim:**

To evaluated the prevalence and potential risk factors of self-reported diabetes among the elderly in China, by demographic data, socioeconomic factors, and psychological factors.

**Methods:**

Descriptive analysis and Chi-square analysis were used to assess the prevalence and variation between self-reported diabetes and non-diabetes by demographic data, living habits, socioeconomic factors and comorbidities. Univariate and multivariate logistic regression were used to describe the odds ratios (OR) of diabetes prevalence in different groups, while stratification analysis was performed to describe prevalence based on gender, age, and urban/rural areas.

**Results:**

215,041 elderly adults (102,692 males and 112,349 females) were eventually included in the analysis. The prevalence of self-reported diabetes among the elderly in China is about 8.7%, with the highest prevalence in Beijing (20.8%) and the lowest prevalence in Xizang (0.9%). Logistic regression analysis showed that urban area (*P* < 0.001), older age (65–84 years old, *P* < 0.001), female (*P* < 0.001), higher income(*P* < 0.001), poor sleep quality (*P* = 0.01) and some other factors were potential risk factors for diabetes.

**Conclusions:**

This study illustrates the prevalence and potential risk factors of diabetes among the elderly in China Meanwhile, these results provide information to assist the government in controlling non-communicable diseases in the elderly.

## Introduction

A growing aging population in China is one of the key challenges facing public healthcare in the country. In fact, China has already become an aging society. In 2019, there were 164.5 million citizens aged 65 or older, including 26 million aged 80 years or more (1). It is predicted that the total population will reach 1.40–1.44 billion by 2030 and 1.29–1.40 billion by 2050 according to a study by the Chinese Center for Disease Control (CDC). The proportion of elderly individuals aged 65 years or more was continuously increasing, from 6.96% in 2000 to 8.87% in 2010, and 13.50% in 2020; this age group will comprise 20% of the total population by 2033 and 30% by 2050 from the Fifth Population Census to the Seventh Population Census (2).

Meanwhile, age-related diseases such as diabetes and complications will impose a significant burden on family and public healthcare systems (3). In 2019, it was estimated that 19.3% of people aged 65–99 years (135.6 million, 95% confidence interval (CI): 107.6–170.6 million) live with diabetes globally. Over the next two decades, the number of people with diabetes will grow from 195.2 million to 276.2 million worldwide (4). In 2015, diabetes cost US$ 1.31 trillion (95% CI 1.28–1.36), or 1.8% (95% CI 1.8–1.9) of the global economy (5). China has experienced a dramatic increase in diabetes prevalence, (6), from 2.5% in 1994 to 9.7% in 2008, and to 11.6% in 2010 (7–9). As of 2019, there are 116.4 million adults in China with diabetes, representing approximately 12.8% of its adult population (10, 11). Approximately USD 165 billion will be spent on diabetes-related health care in 2021, USD 185 billion in 2035, and USD 193 billion in 2045 (11).

The biopsychosocial model was proposed by Engel (12). Cultural, social, and psychological factors are linked with people's health (12). It is known that living conditions and lifestyle are important variables influencing the onset and progress of diabetes. However, previous studies about the prevalence of diabetes had several limitations. First, Most of these studies are regional studies carried out in east China, North China or South China (13–16). Second, the prevalence of diabetes in the general population has been studied more than in the elderly (17). Thirdly, most of the studies have only focused on the biological model of patients (18, 19). And there is little detailed information about socioeconomic factors and psychological factors. For governmental precision medical policies, it is extremely important to find out the comprehensive situation of diabetes patients.

In recent studies, some socioeconomic, lifestyle, and metabolic factors have been identified as risk factors for diabetes (20–22). From an another perspective, this study comprehensively evaluated the prevalence of diabetes among elderly Chinese patients by biomedical factors (age, gender, smoking, alcohol consumption, sleep quality, exercise, comorbidities) and social-psychological factors (education level, marital status, living alone, medical insurance, gainful employment, economic status, and spiritual and cultural life) to find out the awareness and potential risk factors among elderly diabetes patients.

## Methods

### Study design and participants

Government-affiliated China Research Center on Aging is one of China's leading aging research institutions. China Research Centre on Aging initiated the Sample Survey of the Aged Population in Urban and Rural China (SSAPUR) project in 2000. During the survey, the socioeconomic and health characteristics of elderly people over the age of 60 were investigated. This major national condition survey of the elderly in China was followed by longitudinal surveys in 2006 and 2010, the sample size was expanded and resampled in 2015 by Office of the China National Committee on Aging. All four surveys used a similar research design. The 2000 survey included 18,987 observations, the 2006 survey included 18,458 observations, and the 2010 survey included 18,689 observations.

The present study is based on data from the fourth SSAPUR, which was an extensive and large investigation survey of elderly people in China (comprising individuals aged ≥60 years, who were permanent residents and nationally representative; the survey was carried out for 1 month, from 1–31 August 2015. This investigation adopted a stratified multistage and probability proportional to size (PPS) sampling method, with regional sample sizes selected according to the area's proportion of people aged 60 years or more. The first time such a large number has been collected, including all provinces, autonomous regions, municipalities, and Xinjiang Production and Construction Corps across the country, covering 466 counties (districts), 1864 townships (sub-districts), and 7,456 village (residential) committees. The SSAPUR is China's largest elderly population database.

The fourth SSAPUR questionnaire was a large-scale epidemiological survey, which had been used in the Global Burden of Disease study and the World Health Survey. The survey covered nine aspects, including basic demographic information, family status, health, care and nursing services, economic status, social participation, rights protection, livable environment, and spiritual and cultural life (including psychology). Details of the fourth SSAPUR study design and the sampling method are provided in the Supplemental material (Supplements 1, 2).

The research protocol has been approved by the Ethical Review Committee of Beijing Hospital (No.2021BJYYEC-294-01) and approved by National Bureau of Statistics (No [2014] 87). All participants have provided written informed consent.

### Date collection

All data were collected by trained study personnel in accordance with standardized protocols. On the cover of each questionnaire, there was a unique number, start and end time, and the signatures of the surveyors. Due to the huge amount of information gathered, we removed unnecessary information, such as commuting mode and children's work, according to the research purpose, and retained the demographic characteristics, health, social participation, family lives, and psychological information.

At baseline, demographic characteristics included gender, age, education level, household registration, and marital status. The “age” field was filled in, either according to an individual's ID card in the first instance or based on interviews with the senior citizen or his/her family members if he/she did not have an ID card. “Household registration” refers to agricultural household registration or non-agricultural household registration, either written in the household register or determined by the investigators. Education level was classified as follows: uneducated (never received school education at any level or of any type provided by the state or other institutions running schools); primary education (highest level of education received was primary school, whether in school, graduated, or dropped out); junior high-school education (highest level of education received was junior high-school, whether in school, graduated, or dropped out); high-school education (highest level of education received by a person, whether in school, graduated, or dropped out, including general high school, vocational high school, or secondary professional school); junior college (highest level of education received was at junior college); bachelor's degree or above (highest level of education received was a bachelor's degree or above).

Smoking was categorized as never smoked and other situations (including former and current smokers). Alcohol consumption was categorized as never or occasionally, 1–2 times a week, at least three times a week, or often drunk. Sleep quality divided by sleep time cannot describe sleep quality well. In this study, Sleep quality was categorized as very good, relatively good, average, relatively poor, very poor which sorted by elderly themselves. Exercise refers to all types of physical activities that are carried out consciously for the purpose of fitness, but does not include housework or farming. Medical insurance refers to the components of China's medical insurance system (basic medical insurance for urban workers, basic medical insurance for urban residents, and new rural cooperative medical care) and any other medical insurance. Gainful employment refers to the interviewed elderly people who were actually engaged in various production, management, or service activities to earn wages before the survey. Poverty was defined as having an annual household income of <6,000 yuan (US$ 963) in the previous year (2014). Economic status was selected according to the self-rating criteria of the interviewed elderly individuals. Public benefit activities cover safeguarding community public order, helping to mediate neighborhood disputes, safeguarding the community health environment, helping neighbors, caring about educating the next generation (not including educating your own grandchildren), and participating in cultural and scientific promotion activities. Spiritual and cultural life includes watching TV/listening to the radio, reading books/ newspapers, going to the cinema or the theater, Tai Chi/health exercises, playing gateball/table tennis/badminton, or playing mahjong/cards/chess. Chronic diseases, including malignant tumor, cataract/glaucoma, hypertension, cardiac–cerebral vascular disease (CCVD), osteoarthrosis, and chronic obstructive pulmonary disease (COPD), and were self-reported by the interviewed elderly individuals.

### Definition

As a cross-sectional study, the prevalence of diabetes among elderly in this study include those diagnosed by health professionals in the past regardless of subtype (Type 1, Type 2 or any other subtypes), either on diet control, oral hypoglycemic drugs and/or injective insulin. Other chronic diseases which were used as independent variable factors, including malignant tumor, cataract/glaucoma, hypertension and so on, refer to self-reported diseases that has been definitively diagnosed by health professionals before.

### Statistical analysis

From a total of 224,142 cases, we excluded those with missing data, including 9,084 cases whose diabetes status was not clear and 17 individuals who had more than 10 missing independent variables.

The prevalence of diabetes was described by province, in descending order from largest to smallest. Baseline characteristics and other factors were summarized as numbers with proportions. The statistical significance of differences was assessed using Chi-square analysis for categorical variables and a *post hoc* two-tailed Newman–Keuls test when two or more groups were compared.

In univariate logistic regression analysis, demographic data (household registration, age, gender and education level), living habits (smoking, alcohol consumption, sleep quality and exercise), socioeconomic factors (medical insurance, gainful employment, poverty, economic status, public benefit activities, spiritual cultural life), and comorbidities (malignant tumor, cataract/glaucoma, hypertension, CCVD, COPD) were analyzed as independent variables. A *P-*value < 0.05 was considered statistically significant. After that, statistically significant independent variables in the univariate logistic regression analysis were included in the multivariate regression analysis. Stratification analysis was also performed, based on gender, age, and residing in urban or rural areas.The prevalence of diabetes after stratification was tested by chi-square test. A *P-*value <0.05 was considered statistically significant. Using the methods of Robert Newcombe, the lower and upper limits of the 95% confidence intervals for the proportion of diabetics were calculated. All statistical analyses were performed using SPSS 24.0 (IBM Corp., Armonk, NY, USA).

## Results

**Figure 1:**
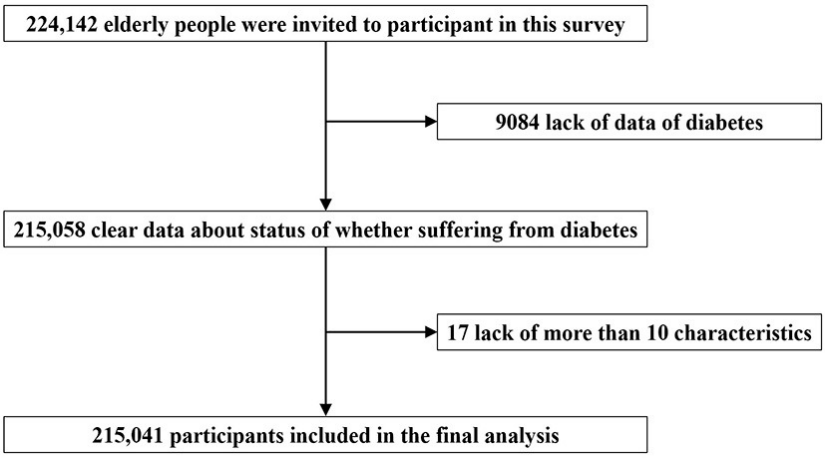
Flowchart of participants on self-reported diabetes of the fourth SSAPUR study.

**Figure 2:**
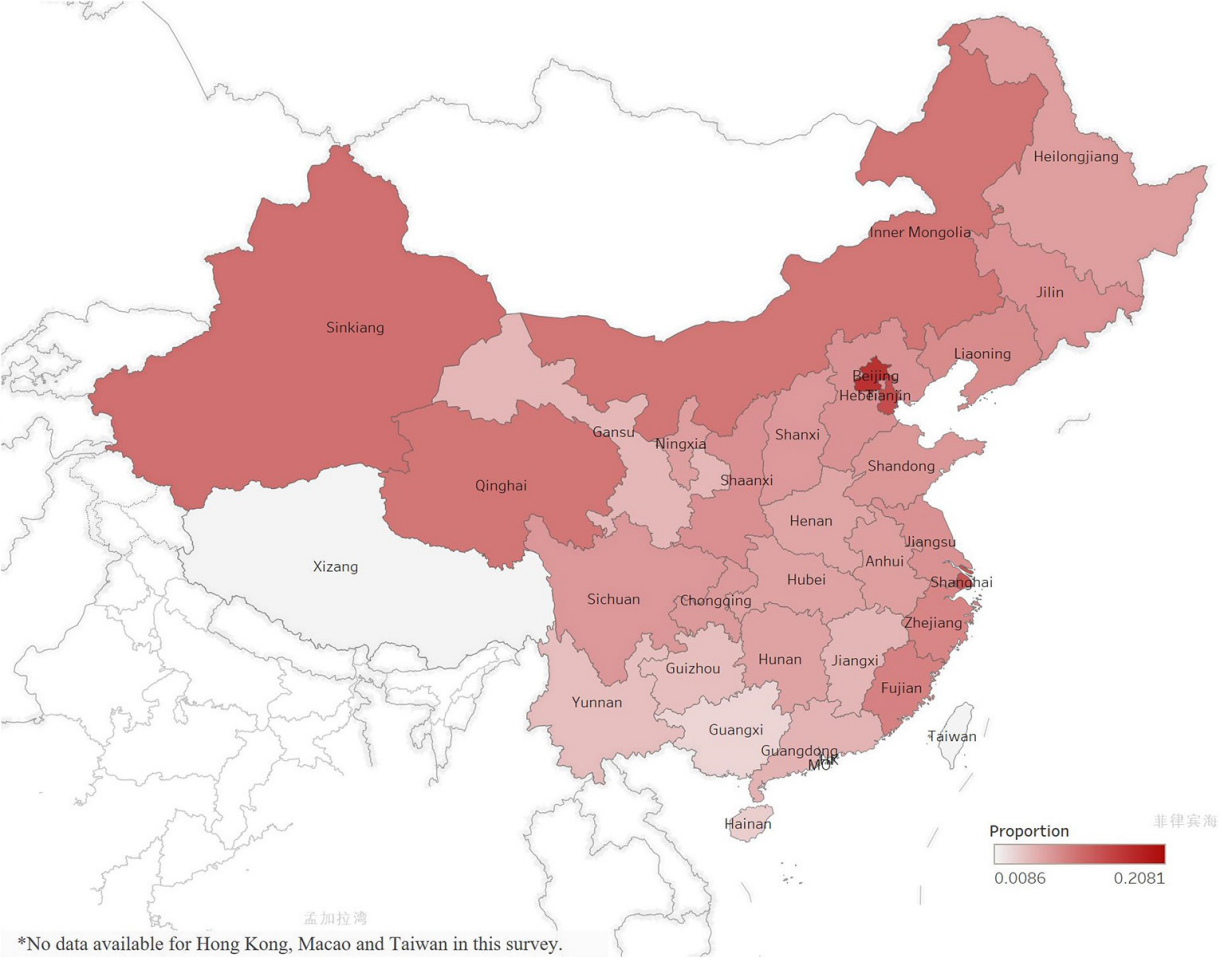
Geographic distribution of diabetes prevalence in 31 selected provinces/municipalities in the fourth SSAPUR study. Hong Kong, Macao and Taiwan were not included in this survey.

Finally, a total of 215,041 participants (102,692 male and 112,349 female) were included in this analysis (Figure 1). Table 1 shows the number of participants and prevalence of diabetes among the elderly in 31 provinces in China. Hong Kong, Macao and Taiwan were not included in this survey. The prevalence of diabetes is 8.7% among the elderly in China, with the highest prevalence in Beijing (20.8%), Tianjin (17.2%), and Shanghai (16%), and the lowest prevalence in Xizang (0.9%), Guangxi (3.4%), and Hainan (3.9%). Figure 2 shows the distribution of the prevalence of diabetes.

**Table 1:** The prevalence of diabetes in the elderly of China.

*Province	**Participants (proportion)**	**Diabetes**	
		**No (proportion)**	**Yes (proportion)**
Total	215,041(100%)	196,319(91.3%)	18,722(8.7%)
Beijing	3,359 (1.6%)	2,660(79.2%)	699(20.8%)
Tianjin	1,920 (0.9%)	1,589(82.8%)	331(17.2%)
Shanghai	4,296 (2.0%)	3,607(84.0%)	689(16.0%)
Sinkiang	2,378 (1.1%)	2,068(87.0%)	310(13.0%)
Qinghai	957 (0.4%)	838(87.6%)	119(12.4)
Inner Mongolia	3,343 (1.6%)	2,930(87.6%)	413(12.4%)
Fujian	5,247 (2.4%)	4,667(88.9%)	580(11.1%)
Zhejiang	9,595 (4.5%)	8,574(89.4%)	1,021(10.6%)
Liaoning	8,573 (4.0%)	7,711(89.9%)	862(10.1%)
Shaanxi	5,754 (2.7%)	5,195(90.3%)	559(9.7%)
Hebei	10,701 (5.0%)	9,683(90.5%)	1,018(9.5%)
Jiangsu	15,629 (7.3%)	14,150(90.5%)	1,479(9.5%)
Jilin	4,222 (2.0%)	3,825(90.6%)	397(9.4%)
Shandong	17,718 (8.2%)	16,134(91.1%)	1,584(8.9%)
Sichuan	16,150 (7.5%)	14,713(91.1%)	1 437(8.9%)
Shanxi	5,250 (2.4%)	4,786(91.2%)	464(8.8%)
Chongqing	6,225 (2.9%)	5,684(91.3%)	541(8.7%)
Anhui	11,240 (5.2%)	10,307(91.7%)	933(8.3%)
Heilongjiang	5,610 (2.6%)	5,147(91.7%)	463(8.3%)
Ningxia	956 (0.4%)	878(91.8%)	78(8.2%)
Hubei	3,551 (1.7%)	3,272(92.1%)	279(7.9%)
Hunan	11,911 (5.5%)	10,982(92.2%)	929(7.8%)
Henan	14,682 (6.8%)	13,553(92.3%)	1,129(7.7%)
Guangdong	13,350 (6.2%)	12,504(93.7%)	846(6.3%)
Jiangxi	6,214 (2.9%)	5,837(93.9%)	377(6.1%)
Gansu	3,344 (1.6%)	3,145(94.0%)	199(6.0%)
Guizhou	5,705 (2.7%)	5,407(94.8%)	298(5.2%)
Yunnan	6,670 (3.1%)	6,322(94.8%)	348(5.2%)
Hainan	1,432 (0.7%)	1,376(96.1%)	56(3.9%)
Guangxi	8,134 (3.8%)	7,858(96.6%)	276(3.4%)
Tibet	925 (0.4%)	917(99.1%)	8(0.9%)

*Hong Kong, Macao and Taiwan were not included in this survey.

The prevalence distribution of diabetes with related factors is shown in Table 2. In urban areas, diabetes prevalence was significantly higher than in rural areas (11.4 vs. 5.8%, *P* = 0.001). The relationship between age and diabetes exhibited a “spoon-shaped” relationship. The prevalence of diabetes was highest in the 70–74 years age group (9.7%), followed by 7.7% and 7.0% in the groups aged 60–64 and ≥85 years, respectively. Females had a higher prevalence of diabetes than males (9.8 vs. 7.6%, *P* < 0.001). The prevalence of diabetes varied among the elderly with different education levels, from uneducated to bachelor's degree or above, with a gradually increasing trend from 7.4 to 16.2% (*P* < 0.001). The prevalence of diabetes also differed by smoking, alcohol consumption, sleep quality, and exercise. There was a difference between the prevalence of diabetes by self-reported economic status (*P* < 0.001), with the “very generous” having the highest prevalence of diabetes (10.1%) and the “relatively difficult” group having the lowest prevalence (8.2%). Diabetes prevalence did not differ significantly between patients with and without cardio-cerebral vascular disease, osteoarthritis, or COPD. The prevalence of diabetes in the hypertensive group (36.9%) was higher than that in the non-hypertensive group (14.6 vs. 5.2%, *P* < 0.001). Patients with cataract/glaucoma (16%) had a higher combined diabetes rate than those without cataract/glaucoma (12.9 vs. 7.9%, *P* < 0.001). Malignant tumor patients also had a higher prevalence of diabetes (1.1%) (11.2 vs. 8.7%, *P* < 0.001).

**Table 2:** The related factors of proportion with (or without) diagnosed diabetes.

**Factor**	**Total (proportion)**	**Diabetes(proportion)**		***P-*value**
		**No**	**Yes**	
Household registration				<0.001
Urban area	111,940(52.1%)	99,210(88.6%)	12,730(11.4%)	
Rural area	103,101(47.9%)	97,109(94.2%)	5,992(5.8%)	
Age(years)				<0.001
60–64	70,913(33.0%)	65,419(92.3%)	5,494(7.7%)	
65–69	50,723(23.6%)	45,999(90.7%)	4,724(9.3%)	
70–74	35,760(16.6%)	32,300(90.3%)	3,460(9.7%)	
75–79	28,131(13.1%)	25,502(90.7%)	2,629(9.3%)	
80–84	18,426(8.6%)	16,783(91.1%)	1,643(8.9%)	
≥85	11,088(5.2%)	10,316(93.0%)	772(7.0%)	
Gender				<0.001
Female	112,349(52.2%)	101,385(90.2%)	10,964(9.8%)	
Male	102,692(47.8%)	94,934(92.4%)	7,758(7.6%)	
Education level				<0.001
Uneducated	63,102(29.4%)	58,439(92.6%)	4,663(7.4%)	
Primary education	89,059(41.5%)	82,186(92.3%)	6,873(7.7%)	
Junior high school	40,508(18.9%)	36,245(89.5%)	4,263(10.5%)	
High school	15,087(7.0%)	13,250(87.8%)	1,837(12.2%)	
Junior College	4,269(2.0%)	3,609(84.5%)	660(15.5%)	
Bachelor degree or above	2,322(1.1%)	1,946(83.8%)	376(16.2%)	
Marital status				<0.001
Married	155,973(72.5%)	142,133(91.1%)	13,840(8.9%)	
Widowed	54,142(25.2%)	49,539(91.5%)	4,603(8.5%)	
Divorce	1,795(0.8%)	1,630(90.8%)	165(9.2%)	
Never married	3,131(1.5%)	3,017(96.4%)	114(3.6%)	
Live alone				<0.001
No	186,118(86.6%)	169,755(91.2%)	16,363(8.8%)	
Yes	28,923(13.4%)	26,564(91.8%)	2,359(8.2%)	
Smoking				<0.001
No	14,533(6.8%)	13,110(90.2%)	1,423(9.8%)	
Yes	200,508(93.2%)	183,209(91.4%)	17,299(8.6%)	
Alcohol consumption				<0.001
Never or occasionally	211,936(98.6%)	193,394(91.3%)	18,542(8.7%)	
1-2 times a week	849(0.4%)	794(93.5%)	55(6.5%)	
At least 3 times a week	1,970(0.9%)	1,855(94.2%)	115(5.8%)	
Often drunk	286(0.1%)	276(96.5%)	10(3.5%)	
Sleep quality				<0.001
Very good	3,145(1.5%)	2,890(91.9%)	255(8.1%)	
Relatively good	6,531(3.0%)	6,006(92.0%)	525(8.0%)	
Average	200,588(93.3%)	183,141(91.3%)	17,447(8.7%)	
Relatively poor	3,993(1.9%)	3,605(90.3%)	388(9.7%)	
Very poor	784(0.4%)	677(86.4%)	107(13.6%)	
Exercise (per week)				<0.001
Never exercise	105,213(48.9%)	97,919(93.1%)	7,294(6.9%)	
Less than once	9,453(4.4%)	8,623(91.2%)	830(8.8%)	
Once or twice	27,582(12.8%)	25,157(91.2%)	2,425(8.8%)	
Three to five times	26,339(12.2%)	23,702(90.0%)	2,637(10.0%)	
Six times and above	46,454(21.6%)	40,918(88.1%)	5,536(11.9%)	
Medical insurance				<0.001
No	1,960(0.9%)	1,852(94.5%)	108(5.5%)	
Yes	213,081(99.1%)	194,467(91.3%)	18,614(8.7%)	
Gainful employment				<0.001
No	193,528(90.0%)	175,946(90.9%)	17,582(9.1%)	
Yes	21513(10.0%)	20,373(94.7%)	1,140(5.3%)	
Poverty				<0.001
No	185,872(86.4%)	168,859(90.8%)	17,013(9.2%)	
Yes	29,169(13.6%)	27,460(94.1%)	1,709(5.9%)	
Economic status				<0.001
Very generous	2,738(1.3%)	2,462(89.9%)	276(10.1%)	
Relatively ample	31,721(14.8%)	28,815(90.8%)	2,906(9.2%)	
Basically enough	126,650(58.9%)	115,657(91.3%)	10,993(8.7%)	
Tougher	45,135(21.0%)	41,429(91.8%)	3,706(8.2%)	
Very difficult	8,797(4.1%)	7,956(90.4%)	841(9.6%)	
Public benefit activities				<0.001
No	117,267(54.5%)	106,276(90.6%)	10,991(9.4%)	
Yes	97,774(45.5%)	90,043(92.1%)	7,731(7.9%)	
Spiritual cultural life				<0.001
No	16,892(7.9%)	15,773(93.4%)	11,191(6.6%)	
Yes	198,149(92.1%)	180,546(91.1%)	17,603(8.9%)	
Malignant tumor				<0.001
No	212,578(98.9%)	194,131(91.3%)	18,447(8.7%)	
Yes	2,463(1.1%)	2,188(88.8%)	275(11.2%)	
Cataract/glaucoma				<0.001
No	180,626(84.0%)	166,343(92.1%)	14,283(7.9%)	
Yes	34,415(16.0%)	29,976(87.1%)	4,439(12.9%)	
Hypertension				<0.001
No	135,768(63.1%)	128,653(94.8%)	7,115(5.2%)	
Yes	79,273(36.9%)	67,666(85.4%)	11,607(14.6%)	
Cardiac-cerebral vascular disease				0.945
No	159,128(74.0%)	145,270(91.3%)	13,858(8.7%)	
Yes	55,913(26.0%)	51,049(91.3%)	4,864(8.7%)	
Osteoarthrosis				0.515
No	121,140(56.3%)	110,551(91.3%)	10,589(8.7%)	
Yes	93,901(43.7%)	85,768(91.3%)	8,133(8.7%)	
COPD				
No	193,092(89.8%)	176,336(91.3%)	16,756(8.7%)	0.164
Yes	21,949(10.2%)	19,983(91.0%)	1,966(9.0%)	

**Figure 3:**
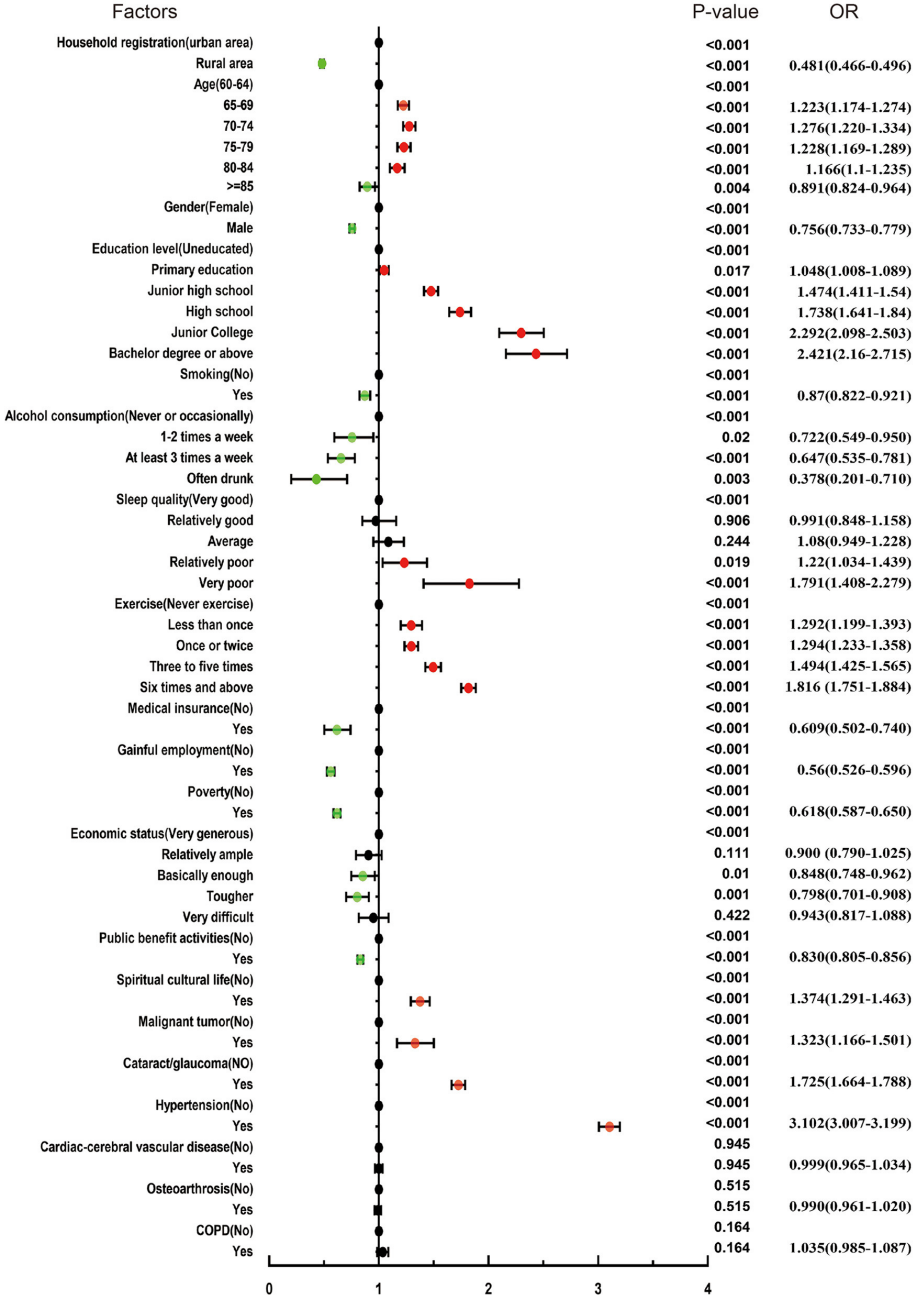
Association between related factors and diabetes by univariable logistic regression analysis.

**Figure 4:**
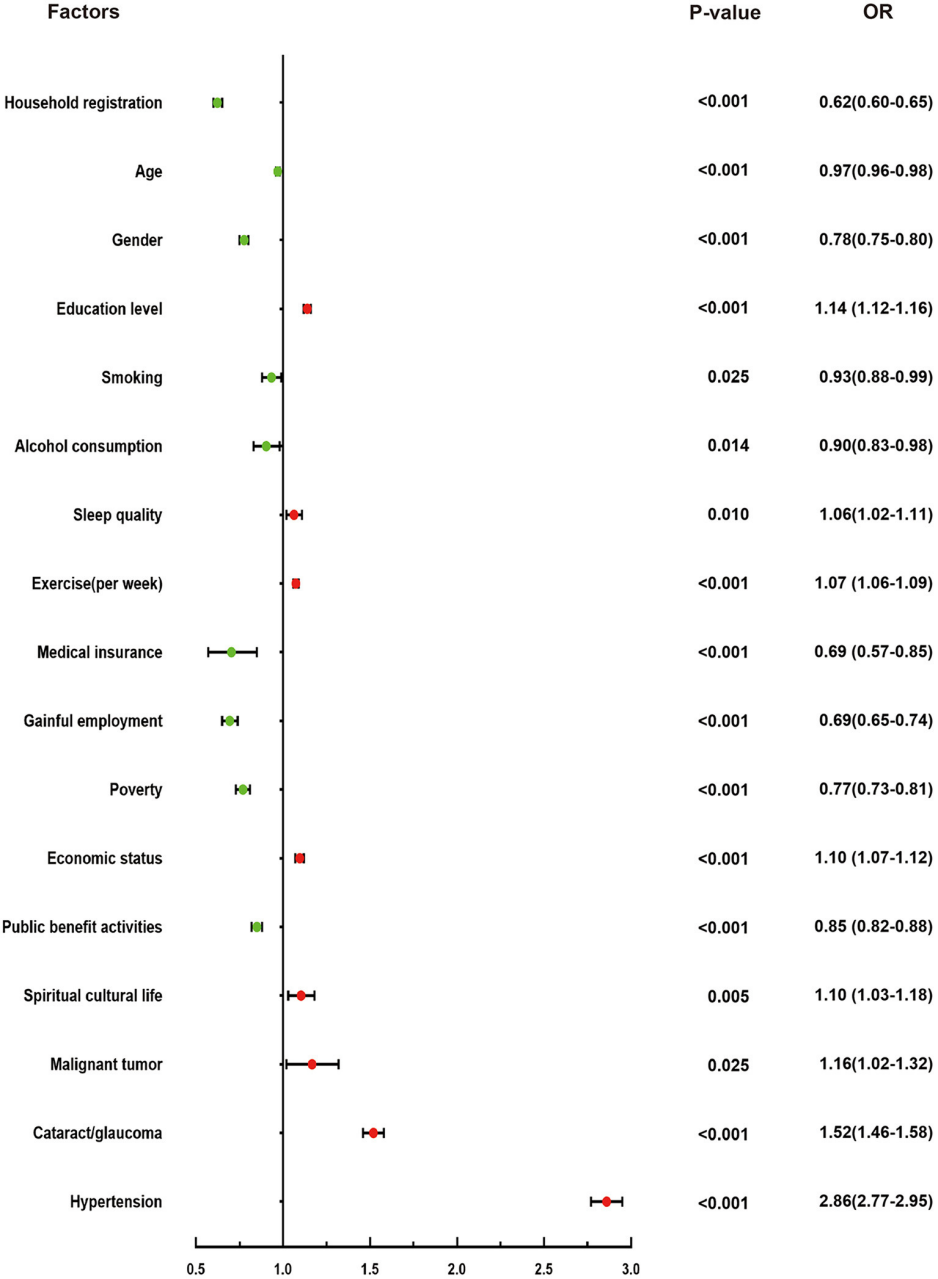
Association between related factors and diabetes by multivariate logistic regression analysis.

Table 3 presents the results of univariate and multivariate logistic regression analysis. Univariate logistic regression showed that the odds ratio (OR) for diabetes in rural areas was 0.48 (95% confidence interval (CI): 0.47–0.50, *P* < 0.001) compared with urban areas. The ORs of diabetes in all age groups increased and then decreased, with the OR of diabetes in the 75–79 years group being 1.23 (95%CI: 1.17–1.29, *P* < 0.001)compared with the 60–64 years group, and the OR of diabetes in the group aged ≥85 years being 0.89 (95%CI: 0.82–0.96, *P* < 0.001). The OR for diabetes in men was 0.76 (95%CI: 0.73–0.78, *P* < 0.001) based on women. The risk of diabetes increased with higher education level. Compared with non-smokers, smokers had a lower prevalence of diabetes.The OR for “very poor” sleep quality was 1.79 (95%CI: 1.41–2.28, *P* < 0.001) compared with “very good” sleep quality. The odds of diabetes among the elderly from poor families with an annual household income <6,000 yuan (US$ 963) was 0.62 times that of non-poor families (95%CI: 0.59–0.65, *P* < 0.001). Those with malignant tumors (OR: 1.32, 95%CI: 1.17–1.50, *P* < 0.001), cataract/glaucoma (OR: 1.73, 95%CI: 1.66–1.79, *P* < 0.001), and high blood pressure (OR: 3.10, 95%CI: 3.01–3.20, *P* < 0.001) showed a higher prevalence of diabetes than people without these diseases. In this study, the proportion of diabetes patients with CCVD or osteoarthrosis was not statistically significant compared with the proportion of people without such diseases (Figure 3). In multivariate logistic regression analysis, the statistically significant factors were included. The results are presented in Table 3 and they were all statistically significant (Figure 4).

**Table 3:** Univariate and multivariate logistic analysis for diabetes.

**Factor**	**Univariate**		**Multivariate**	
	**OR(95%CI)**	***P-*value**	**OR(95%CI)**	***P-*value**
Household registration		<0.001	0.62(0.60–0.65)	<0.001
Urban area	1		1	
Rural area	0.48(0.47–0.50)	<0.001		
Age(years)		<0.001	0.97(0.96–0.98)	<0.001
60-64	1		1	
65-69	1.22 (1.17–1.27)	<0.001		
70-74	1.28(1.22–1.33)	<0.001		
75-79	1.23(1.17–1.29)	<0.001		
80-84	1.17(1.10–1.24)	<0.001		
≥85	0.89(0.82–0.96)	0.004		
Gender		<0.001	0.78(0.75–0.80)	<0.001
Female	1		1	
Male	0.76(0.73–0.78)	<0.001		
Education level		<0.001	1.14 (1.12–1.16)	<0.001
Uneducated	1		1	
Primary education	1.05 (1.01–1.09)	0.017		
Junior high school	1.47(1.41–1.54)	<0.001		
High school	1.74(1.64–1.84)	<0.001		
Junior College	2.29(2.10-2.50)	<0.001		
Bachelor degree or above	2.42(2.16-2.72)	<0.001		
Smoking		<0.001	0.93(0.88–0.99)	0.025
No	1		1	
Yes	0.87(0.82–0.92)	<0.001		
Alcohol consumption		<0.001	0.90(0.83–0.98)	0.014
Never or occasionally	1		1	
1–2 times a week	0.72(0.55–0.95)	0.02		
At least 3 times a week	0.65(0.54–0.78)	<0.001		
Often drunk	0.38(0.20–0.71)	0.003		
Sleep quality		<0.001	1.06(1.02–1.11)	0.010
Very good	1		1	
Relatively good	0.99(0.85–1.16)	0.906		
Average	1.08(0.95–1.23)	0.244		
Relatively poor	1.22(1.03–1.44)	0.019		
Very poor	1.79(1.41-2.28)	<0.001		
Exercise(per week)		<0.001	1.07 (1.06–1.09)	<0.001
Never exercise	1		1	
Less than once	1.29(1.20–1.39)	<0.001		
Once or twice	1.29(1.23–1.36)	<0.001		
Three to five times	1.49(1.43–1.57)	<0.001		
Six times and above	1.82 (1.75–1.88)	<0.001		
Medical insurance			0.69 (0.57–0.85)	<0.001
No	1		1	
Yes	0.61(0.50–0.74)	<0.001		
Gainful employment			0.69(0.65–0.74)	<0.001
No	1		1	
Yes	0.56(0.53–0.60)	<0.001		
Poverty			0.77(0.73–0.81)	<0.001
No	1		1	
Yes	0.62(0.59–0.65)	<0.001		
Economic status			1.10 (1.07–1.12)	<0.001
Very generous	1		1	
Relatively ample	0.90(0.79–1.03)	0.111		
Basically enough	0.85(0.75–0.96)	0.010		
Tougher	0.80 (0.70–0.91)	0.001		
Very difficult	0.94(0.82–1.09)	0.422		
Public benefit activities			0.85 (0.82–0.88)	<0.001
No	1		1	
Yes	0.83(0.81–0.86)	<0.001		
Spiritual cultural life			1.10 (1.03–1.8)	0.005
No	1		1	
Yes	1.37 (1.29–1.46)	<0.001		
Malignant tumor			1.16(1.02–1.32)	0.025
No	1		1	
Yes	1.32(1.17–1.50)	<0.001		
Cataract/glaucoma			1.52(1.46–1.58)	<0.001
No	1		1	
Yes	1.73(1.67–1.79)	<0.001		
Hypertension			2.86(2.77-2.95)	<0.001
No	1		1	
Yes	3.10(3.01-3.20)	<0.001		
Cardiac-cerebral vascular disease				
No	1			
Yes	1.00(0.97–1.03)	0.945		
Osteoarthrosis				
No	1			
Yes	1.00(0.96–1.02)	0.515		
COPD				
No	1			
Yes	1.04(0.99–1.09)	0.164		

By stratifying by gender, we explored the prevalence and differences between parameters and diabetes (Table 4). A total of 10,964 female individuals with diabetes were identified among 112,349 female participants. Diabetes prevalence varied between urban and rural areas among females (12.0 vs. 7.2%, *P* < 0.001) and among age groups (*P* < 0.001). In addition, a total of 7,758 male individuals with diabetes were identified among 102,692 male participants. Among males, the prevalence of diabetes varied throughout urban and rural areas (10.7 vs. 4.3%, *P* < 0.001) and among age groups (*P* < 0.001). Regardless of gender, the prevalence of diabetes differed by comorbidities such as cataract glaucoma (*P* < 0.001) and hypertension (*P* < 0.001). However, The prevalence of diabetes in women with or without COPD was significantly different (12.8 vs. 9.7%, *P* < 0.001), but this was not seen in men (7.5 vs. 7.6%, *P* = 0.728). In males with and without osteoarthrosis, prevalence of diabetes varied significantly (7.1 vs. 7.8%, *P* < 0.001), but not in females (9.8 vs. 9.8%, *P* = 0.971).

**Table 4:** The prevalence of diabetes by gender/urban and rural/age stratification.

**Factors**	**Gender**		**Age**			**Urban and Rural area**	
	**Female**	**Male**	**60–69**	**70–79**	**≥80**	**Urban area**	**Rural area**
**Gender**							
Female			5,947(9.6%)	3,626(10.8%)	1,391(8.2%)	7,160(12%)	3,804(7.2%)
Male			4,271(7.1%)	2,463(8.1%)	1,024(8.2%)	5,570(10.7%)	2,188(4.3%)
*P-*value			<0.001	<0.001	0.943	<0.001	<0.001
**Age(years)**							
60-64	3,187(8.8%)	2,307(6.6%)				3,502(9.7%)	1,992(5.7%)
65-69	2,760(10.7%)	1,964(7.9%)				3,148(12.2%)	1,576(6.3%)
70-74	2,016(10.9%)	1,444(8.4%)				2,392(12.9%)	1,068(6.2%)
75-79	1,610(10.8%)	1,019(7.7%)				1,878(12.6%)	751(5.7%)
80-84	965(9.4%)	678(8.4%)				1,237(11.9%)	406(5.0%)
≥85	426(6.4%)	346(7.9%)				573(9.4%)	199(4.0%)
*P-*value	<0.001	<0.001				<0.001	<0.001
**Household registration**							
Urban area	7,160(12.0%)	5,570(10.7%)	6,650(10.7%)	4,270(12.8%)	1,810(11.0%)		
Rural area	3,804(7.2%)	2,188(4.3%)	3,568(6.0%)	1,819(6.0%)	605(4.6%)		
*P-*value	<0.001	<0.001	<0.001	<0.001	<0.001		
**Education level**							
Uneducated	3,987(8.3%)	676(4.6%)	1,971(7.6%)	1,655(7.8%)	1,037(6.5%)	2,350(9.6%)	2,313(6%)
Primary education	4,120(9.8%)	2,753(5.8%)	3,992(7.3%)	2,183(8.5%)	698(7.9%)	4,185(10.2%)	2,688(5.6%)
Junior high school	1,828(12.7%)	2,435(9.3%)	2,732(9.6%)	1,208(12.2%)	323(14.1%)	3,450(12.9%)	813(5.9%)
High school	749(13.5%)	1,088(11.4%)	999(10.8%)	651(14.6%)	187(13.9%)	1,686(13.3%)	151(6.3%)
Junior College	174(14.1%)	486(18%)	360(14.5%)	207(16.3%)	93(18.1%)	651(15.6%)	9(10.7%)
Bachelor degree or above	81(12.9%)	295(17.4%)	137(16.1%)	168(16.5%)	71(15.8%)	375(16.2%)	1(12.5%)
*P-*value	<0.001	<0.001	<0.001	<0.001	<0.001	<0.001	0.036
**Marital status**							
Married	7,200(10.0%)	6,640(7.9%)	8,717(8.5%)	4,089(9.8%)	1,034(9.2%)	9,485(11.5%)	4,355(5.9%)
Widowed	3,675(9.3%)	928(6.3%)	1,295(8.4%)	1,936(9.3%)	1,372(7.7%)	3,067(11.3%)	1,536(5.7%)
Divorce	83(11.8%)	82(7.5%)	128(9.2%)	34(10.7%)	3(3.4%)	135(10.4%)	30(6%)
Never married	6(3.4%)	108(3.7%)	78(4%)	30(3.1%)	6(2.8%)	43(4.5%)	71(3.2%)
*P-*value	<0.001	<0.001	<0.001	<0.001	<0.001	<0.001	<0.001
**Live alone**							
No	9,297(9.8%)	7,066(7.8%)	9,394(8.5%)	5,051(9.6%)	1,918(8.6%)	11,241(11.5%)	5,122(5.8%)
Yes	1,667(9.6%)	692(6%)	824(7.8%)	1,038(9.2%)	497(7%)	1,489(10.8%)	870(5.8%)
*P-*value	0.482	<0.001	0.019	0.213	<0.001	0.021	0.741
**Smoking**							
No	1,056(9.9%)	367(9.5%)	762(9.5%)	457(10.9%)	204(8.8%)	978(12.4%)	445(6.7%)
Yes	9,908(9.7%)	7,391(7.5%)	9,456(8.3%)	5,632(9.4%)	2,211(8.1%)	11,752(11.3%)	5,547(5.8%)
*P-*value	0.649	<0.001	<0.001	0.001	0.239	0.002	0.002
Alcohol consumption							
Never or occasionally	10,946(9.8%)	7,596(7.6%)	10,104(8.4%)	6,039(9.6%)	2,399(8.2%)	12,615(11.4%)	5,927(5.8%)
1–2 times a week	10(8.1%)	45(6.2%)	40(6.9%)	12(5.8%)	3(4.6%)	36(8.9%)	19(4.3%)
At least 3 times a week	8(4.3%)	107(6%)	67(5.3%)	36(7%)	12(6.2%)	73(7.9%)	42(4%)
Often drunk	0(0%)	10(3.8%)	7(3.6%)	2(3.2%)	1(3.1%)	6(4.9%)	4(2.4%)
*P-*value	0.03	0.003	<0.001	0.016	0.355	<0.001	0.009
**Sleep quality**							
Very good	108(8.7%)	147(7.7%)	141(7.6%)	80(9.1%)	34(8.1%)	179(9.8%)	76(5.8%)
Relatively good	276(9.2%)	249(7.0%)	300(7.8%)	157(8.9%)	68(7.3%)	396(11%)	129(4.4%)
Average	10,230(9.7%)	7,217(7.6%)	9,516(8.4%)	5,682(9.5%)	2,249(8.2%)	11,851(11.4%)	5,596(5.8%)
Relatively poor	270(10.6%)	118(8.1%)	205(9.8%)	134(10.7%)	49(7.7%)	245(13.6%)	143(6.5%)
Very poor	80(14.5%)	27(11.5%)	56(13.5%)	36(14.5%)	15(12.5%)	59(15.3%)	48(12%)
*P-*value	0.001	0.111	<0.001	0.038	0.392	0.001	<0.001
**Exercise**							
Never exercise	4,774(8.3%)	2,520(5.3%)	3,641(6.5%)	2,370(7.6%)	1,283(7.3%)	3,760(9.6%)	3,534(5.4%)
Less than once	533(10.3%)	297(7.0%)	397(7.9%)	293(10.3%)	140(8.8%)	615(11.7%)	215(5.1%)
Once or twice	1,437(9.9%)	988(7.6%)	1,362(8.3%)	788(9.9%)	275(8.6%)	1,711(10.9%)	714(6%)
3–5five times	1,490(11.1%)	1,147(8.9%)	1,597(9.9%)	801(10.5%)	239(9.5%)	1,985(11.7%)	652(6.9%)
Six times and above	2,730(12.6%)	2,806(11.3%)	3,221(11.6%)	1,837(13%)	478(10.5%)	4,659(13.4%)	877(7.6%)
*P-*value	<0.001	<0.001	<0.001	<0.001	<0.001	<0.001	<0.001
**Medical insurance**							
No	10,886(9.8%)	7,728(7.6%)	10,155(8.4%)	6,057(9.6%)	2,402(8.2%)	12,654(11.4%)	5,960(5.8%)
Yes	78(7.2%)	30(3.4%)	63(5.9%)	32(6.1%)	13(3.6%)	76(7.6%)	32(3.3%)
*P-*value	0.004	<0.001	0.003	0.008	0.002	<0.001	0.001
**Gainful employment**							
No	10,556(10.0%)	7,026(8%)	9,250(9%)	5,936(9.7%)	2,396(8.2%)	11,998(11.9%)	5,584(6%)
Yes	408(6.4%)	732(4.8%)	968(5.2%)	153(5.5%)	19(7.1%)	732(6.8%)	408(3.8%)
*P-*value	<0.001	<0.001	<0.001	<0.001	0.512	<0.001	<0.001
**Poverty**							
No	9,801(10.2%)	7,212(8.0%)	9,400(8.6%)	5,423(10.3%)	2,190(9.1%)	12,186(11.7%)	4,827(5.9%)
Yes	1,163(7.2%)	546(4.2%)	818(6.5%)	666(6.0%)	225(4.2%)	544(6.8%)	1,165(5.5%)
*P-*value	<0.001	<0.001	<0.001	<0.001	<0.001	<0.001	0.039
**Economic status**							
Very generous	132(10.4%)	144(9.8%)	147(9.5%)	84(10.6%)	45(11%)	229(12.1%)	47(5.6%)
Relatively ample	1,467(9.5%)	1,439(8.9%)	1,516(8.1%)	957(11%)	433(10.3%)	2,313(11.2%)	593(5.3%)
Basically enough	6,422(9.7%)	4,571(7.6%)	6,054(8.3%)	3,554(9.6%)	1,385(8.2%)	7,899(11.5%)	3,094(5.4%)
Tougher	2,416(9.9%)	1,290(6.2%)	2,040(8.5%)	1,212(8.3%)	454(6.9%)	1,883(10.9%)	1,823(6.5%)
Very difficult	527(11.2%)	314(7.7%)	461(10.4%)	282(9.6%)	98(6.8%)	406(12.5%)	435(7.9%)
*P-*value	0.006	<0.001	<0.001	<0.001	<0.001	0.056	<0.001
**Public benefit activities**							
No	6,643(10.3%)	4,348(8.3%)	5,333(9%)	3,786(10.4%)	1,872(8.7%)	7,709(12.1%)	3,282(6.1%)
Yes	4,321(9%)	3,410(6.8%)	4,885(7.8%)	2,303(8.4%)	543(6.9%)	5,021(10.4%)	2,710(5.5%)
*P-*value	<0.001	<0.001	<0.001	<0.001	<0.001	<0.001	<0.001
**Spiritual cultural life**							
No	773(7.1%)	346(5.7%)	392(6.7%)	408(7.3%)	319(5.9%)	543(9.3%)	576(5.2%)
Yes	10,191(10.0%)	7,412(7.7%)	9,826(8.5%)	5,681(9.7%)	2,096(8.7%)	12,187(11.5%)	5,416(5.9%)
*P-*value	<0.001	<0.001	<0.001	<0.001	<0.001	<0.001	0.004
**Malignant tumor**							
No	10,804(9.7%)	7,643(7.5%)	10,080(8.4%)	5,997(9.5%)	2,370(8.1%)	12,507(11.3%)	5,940(5.8%)
Yes	160(12.8%)	115(9.5%)	138(9.7%)	92(12.1%)	45(16.1%)	223(14.3%)	52(5.7%)
*P-*value	<0.001	0.011	0.08	0.014	<0.001	<0.001	0.913
**Cataract/glaucoma**							
No	8,066(8.9%)	6,217(6.9%)	8,409(7.8%)	4,346(8.5%)	1,528(7.1%)	9,492(10.3%)	4,791(5.4%)
Yes	2,898(13.5%)	1,541(11.9%)	1,809(13.3%)	1,743(13.6%)	887(11.2%)	3,238(16.4%)	1,201(8.2%)
*P-*value	<0.001	<0.001	0.03	<0.001	<0.001	<0.001	<0.001
**Hypertension**							
No	3,929(5.8%)	3,186(4.7%)	4,278(5.3%)	2,132(5.7%)	705(4%)	4,779(7.1%)	2,336(3.4%)
Yes	7,035(15.8%)	4,572(13.1%)	5,940(14.6%)	3,957(14.9%)	1,710(14.2%)	7,951(17.8%)	3,656(10.6%)
*P-*value	<0.001	<0.001	<0.001	<0.001	<0.001	<0.001	<0.001
**Cardiac-cerebral vascular disease**							
No	7,944(9.8%)	5,914(7.6%)	7,519(8.3%)	4,501(9.5%)	1,838(8.5%)	9,539(11.3%)	4,319(5.8%)
Yes	3,020(9.7%)	1,844(7.4%)	2,699(8.6%)	1,588(9.6%)	577(7.4%)	3,191(11.4%)	1,673(6%)
*P-*value	0.943	0.275	0.191	0.875	0.002	0.640	0.196
**Osteoarthrosis**							
No	5,567(9.8%)	5,022(7.8%)	5,929(8.4%)	3,376(9.7%)	1,284(7.9%)	7,602(11.3%)	2,987(5.6%)
Yes	5,397(9.8%)	2,736(7.1%)	4,289(8.3%)	2,713(9.3%)	1,131(8.5%)	5,128(11.5%)	3,005(6.1%)
*P-*value	0.971	<0.001	0.504	0.073	0.069	0.203	<0.001
**COPD**							
No	9,900(9.7%)	6,856(7.6%)	9,297(8.4%)	5,390(9.6%)	2,069(8.1%)	11,522(11.3%)	5,234(5.7%)
Yes	1,064(10.8%)	902(7.5%)	921(8.9%)	699(9.1%)	346(9%)	1208(12.1%)	758(6.4%)
*P-*value	<0.001	0.728	0.068	0.136	0.057	0.024	0.007

“Urban and rural” stratification was used to analyze the prevalence and differences between parameters and diabetes (Table 4). Within the 111,940 urban participants, 12,730 individuals had diabetes. The prevalence of diabetes in urban areas varied between females and males (12 vs. 10.7%, *P* < 0.001) and between age groups (*P* < 0.001). Out of 103,101 participants living in urban areas, 5,992 were diagnosed with diabetes. The prevalence of diabetes in rural areas varied between females and males (7.2 vs. 4.3%, *P* < 0.001).

**Figure 5:**
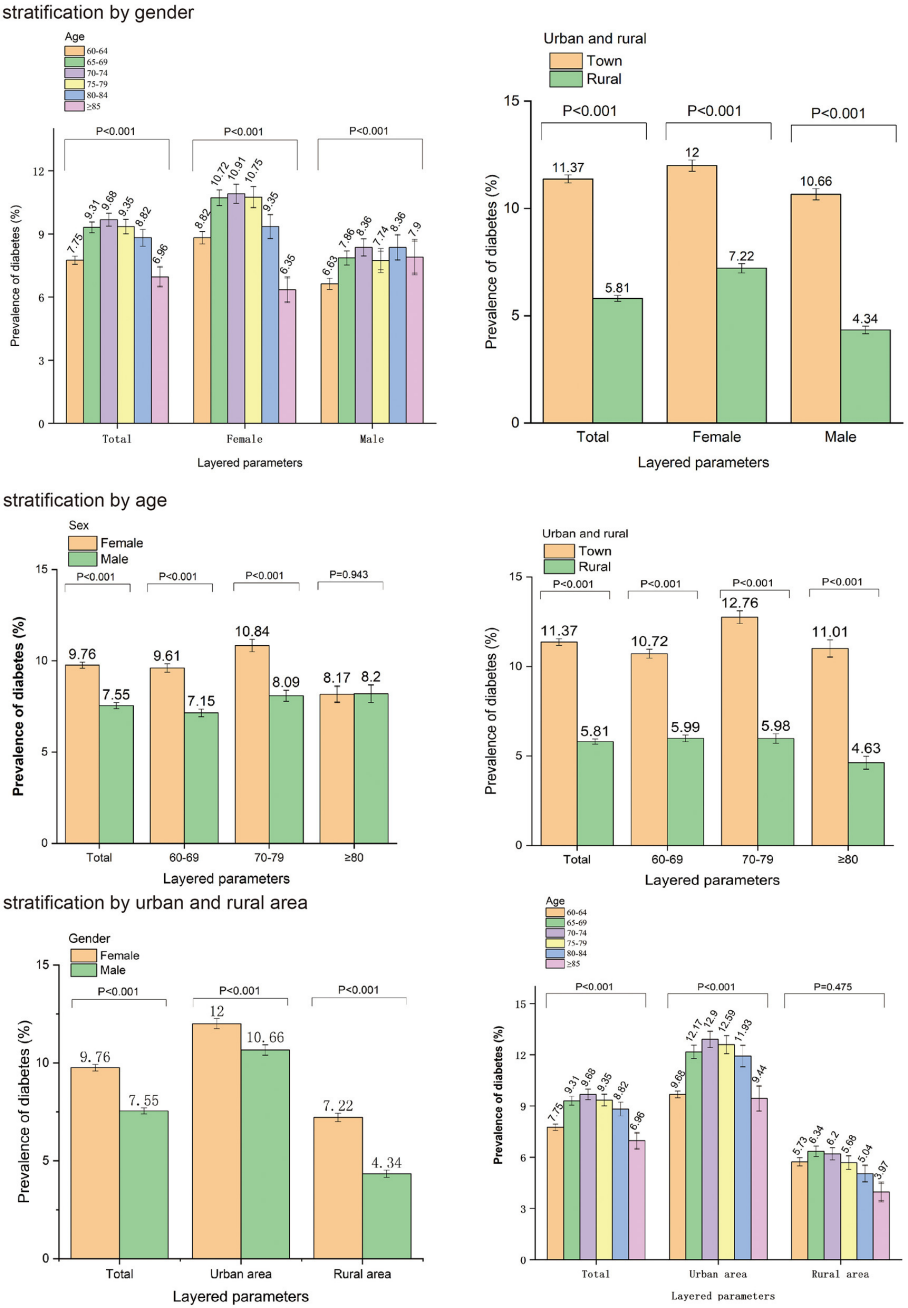
The relationship between parameters and diabetes was further analyzed by gender, age, and urban and rural residence stratification.

An age-specific analysis of the prevalence and differences between parameters and diabetes was further conducted (Table 4). We divided the continuous variable “age” into three groups, spaced at 10 years. A total of 10,218 individuals with diabetes were identified among 121,636 participants aged between 60 and 69 years. The prevalence of diabetes in this age group varied between urban and rural areas (10.7 vs. 6.0%, *P* < 0.001) and between females and males (9.6 vs. 7.1%, *P* < 0.001). The resulting bar chart is shown in Figure 5.

## Discussion

This national cross-sectional study of older adults aged more than 60 years describes the national distribution of self-reported diabetes by demographic data, living habits, socioeconomic factors and comorbidities. A stratified sampling method was used to investigate the elderly people of all provinces in China. The questionnaire has high reliability and validity, and the amount of data is large and reliable. Meanwhile, socioeconomic factors, living habits and comorbidities were included in the analysis as potential risk factors. This further expands our understanding of the causes on diabetes. Further stratified analyses were conducted to illustrate the differences of diabetes and other risk factors by gender, age, urban and rural areas. It can help medical workers to provide targeted prevention and treatment measures.

In this study, the prevalence of self-reported diabetes in China among people aged more than 60 years is 8.7%. Weiqing Wang et al. analyzed data from the China Cardiometabolic Disease and Cancer Cohort Study, which included 93,781 subjects with a mean age of 55.7 years, of whom 67% were women. During a mean follow-up period of about 3 years, 6,171 new cases of diabetes were identified. And the incidence of diabetes was 6.58% based on blood glucose testing (20). In a study of the China Kadoorie Biobank, 8,784 out of 461,211 (prevalence: 19.0%) adults aged 30–79 years were diagnosed with type 2 diabetes during a median follow-up of about 7 years (23). Wang et al. proposed that the self-reported prevalence of diabetes is 8.4% in middle-aged and elderly Chinese (24). In another study of about 10, 000 participants conducted in 2011–2012, the prevalence of self-reported diabetes and screening-detected prevalence was 6.0 and 9.8% among people over 44 years of age (25). Our study was a nationwide cross-sectional study in 2015. Self-reported prevalence of diabetes was slightly higher than the two studies above, probably because of increased aging, physical examination, and adequate nutrition of the elderly. Of those surveyed, 58.7% to 69.9% were unaware of their diagnosis (8, 9, 26). According to the available data, undiagnosed diabetes still accounts for a large proportion of cases, and prediabetes may represent an even larger proportion (27).

Women are more likely than men to suffer from diabetes according to our study. Gender differences in diabetes have varied in previously reported studies (28). However, research has shown that some important risk factors, such as obesity, sex hormones, and psychological stress problems, are more common in women, supporting our finding that older women develop diabetes more frequently than men (29). Our study showed that age is an independent risk factor for diabetes. However, the prevalence of diabetes does not completely increase with age. The prevalence of diabetes is highest in people aged 70–74 years and lowest in people aged more than 85 years. A meta-analysis revealed a prevalence of 11.0% (95% CI 9.0–13.0%) among 55–64-year-olds, 14.1% (95% CI 12.3–16%) among 65–74-year-olds, and 11.0% (95% CI 9.0–13.0%) over 75-year-olds (30). This is similar to the results of our study. These results were obtained because self-reported diagnosis of diabetes in the old may be biased due to cognitive decline and shortened life expectancy in the elderly with diabetic macroangiopathy.

According to our study, diabetes prevalence is higher in economically developed provinces than in less developed ones. Meanwhile, compared to rural areas, urban areas had a higher prevalence of diabetes, according to our survey. As in some earlier geographical studies, the prevalence of diabetes in economically developed provinces and northern provinces was higher than that in economically underdeveloped and central or southern regions (30, 31). A study of 512,869 participants in China indicated that 4.1% of diabetes patients live in rural areas, compared with 8.1% in urban areas (18). It is well known that diabetes is highly related to nutritional status and obesity, and diet structure and lifestyle can affect the incidence of diabetes. At the same time, the medical conditions in the developed areas are better, and people pay more attention to health, so the early detection of diabetes is more likely. The prevalence of diabetes in participants with higher level of education was higher, which is similar to many previously published studies (31–33).

In our study, older adults with diabetes smoked and drank less but exercised often. Smoking and drinking are recognized as unhealthy lifestyles that can increase the risk of diabetes, while exercise is recognized as a healthy lifestyle, so this finding could be interpreted as reflecting good health education and lifestyle interventions of people with diabetes in China. In our study, Poor sleep quality is an independent risk factor for diabetes. And the prevalence of diabetes was significantly higher among relatively poor and very poor sleepers, which could be related to chronic stress stimulation and increased body mass index., The study by Wang et al. (34) shows that obstructive sleep apnea has been linked to abnormal glucose metabolism in laboratory-based experiments. Sleep apnea is highly correlated with poor sleep quality, which may partly explain the relationship between sleep and diabetes. Diabetes patients' sleep duration is also associated with glycemic control.

Interestingly, the never-married group had significantly lower rates of diabetes than the married group. The specific cause of this is unclear and needs further study. We found that 99.1% of Chinese citizens had medical insurance, and the prevalence of diabetes among this group was also higher, which may be related to increased rates of outpatient visits and subsequent diagnoses. Our study also suggests that diabetic patients are more actively involved in spiritual and cultural life than non-diabetic patients. This may benefit from the widespread awareness of lifestyle intervention for diabetes.

People with hypertension were significantly more likely to develop diabetes than those with normal blood pressure. Diabetes and hypertension share numerous pathophysiological mechanisms and genetic factors. Consequently, both clinical entities contribute synergistically to micro- and macro-vasculopathy and cardiovascular death (35). In a study of 318,664 individuals, it was found that T2DM is associated with hypertension, but that the causal relationship is unlikely (36). The prevalence of diabetes in tumor patients and cataract/glaucoma patients was also higher than that in patients without these comorbidities. Diabetes patients may be at a higher risk of cancer due to risk factors such as age, obesity, inactivity, and smoking. Several types of cancer are also affected by diabetes, including hepatocellular cancer, hepatobiliary cancer, pancreatic cancer, ovarian cancer, breast cancer, endometrial cancer, and gastrointestinal cancer. Hyperglycemia, increased bioactivity of insulin-like growth factor 1, hyperinsulinemia, dysregulation of sex hormones, oxidative stress, and chronic inflammation are some of the biological mechanisms linking diabetes and cancer (37). It is recognized that T2DM is a risk factor for cataract development (38). Glaucoma and diabetes share some risk factors and pathophysiologic features, but their pathophysiology is not completely understood. The presence of diabetes and elevated fasting glucose levels is also related to elevated intraocular pressure, which is one of the key risk factors for glaucomatous optic neuropathy (39). Diabetes prevalence was not statistically significant in the elderly with or without COPD, but not among women. A neglected relationship is that of the diabetes–lung association, which is epidemiologically and clinically well-established, including asthma and COPD; however, the underlying mechanism and pathophysiology are not fully understood (40). In our study, there were no statistically significant differences of prevalence of diabetes in the elderly with or without osteoarthritis, but it was not seen among men. The reason for this gender difference is unclear. A meta-analysis of 49 studies found a significant association between osteoarthritis and type 2 diabetes (41). There are two major pathways involved in the pathogenesis of T2DM leading to osteoarthritis: oxidative stress and low-grade chronic inflammation caused by chronic hyperglycemia and insulin resistance (42).

## Limitations

The limitations of this study may include the following aspects. Firstly, this is a cross-sectional study. And potential risk factor analysis is correlation analysis, not causality analysis. Secondly, we do not test blood sugar to distinguish the hidden diabetes and pre-diabetes, nor do we gather information of the control and treatment of diabetes among the elderly. There are biases from the older persons due to recall bias or cognitive impairment. Thirdly, some variables were not evaluated. The dimensions and number of variables were large, and there was a lack of assessment on presence and change of these modifiable lifestyles, before or after the diagnosis of diabetes. Nonetheless, to our knowledge, the present study demonstrated the awareness of diabetes diagnosis, lifestyle and economic status of the Chinese elderly. The study also provided insights into socioeconomic, lifestyle, comorbidities and other potential risk factors for diabetes. These results could be helpful for further research and comprehensive understanding of diabetes.

## Conclusions

With the advent of the aging society in China, the prevalence of diabetes as a disease of aging is increasing. Besides genetic and metabolic factors, socioeconomic factors, living habits, and comorbidities are also potential independent risk factors for diabetes. This study makes us realize that diabetes has a complex pathogenesis involving both environmental and individual factors. Further studies can be conducted based on the results drawn from this study. Diabetes, as one of the diseases with increasing prevalence, and its serious complications have a great impact on the physical and mental health of patients, which needs more attention and financial investment from the government. This study also provides more relevant references for medical administrative departments on diabetes.

## Data availability statement

The original contributions presented in the study are included in the article/Supplementary material, further inquiries can be directed to the corresponding author/s.

## Ethics statement

The research protocol has been approved by the Ethical Review Committee of Beijing Hospital (No. 2021BJYYEC-294-01) and approved by National Bureau of Statistics (No. [2014] 87). All participants have obtained written informed consent. The patients/participants provided their written informed consent to participate in this study.

## Author contributions

DL and JuL: conceived and designed the study. XH and LM: supervised the study. XH, HX, JiL, YL, and NJ: performed statistical analysis. XH, ZW, HL, XQ, XZ, and QZ: drafted the report. All authors revised the report and approved the final version before submission. All authors contributed to data collection, analysis, and interpretation.

## Supplementary material

Supplement 1 Sampling plan of the fourth Sample Survey of the Aged Population in Urban and Rural China (SSAPUR).

Supplement 2 Personal questionnaire of the fourth SSAPUR.
